# The sum of gains and losses of genes encoding the protein tyrosine kinase targets predicts response to multi-kinase inhibitor treatment: Characterization, validation, and prognostic value

**DOI:** 10.18632/oncotarget.4557

**Published:** 2015-07-21

**Authors:** Xiaojun Jiang, Daniel Pissaloux, Christelle De La Fouchardiere, Françoise Desseigne, Qing Wang, Valery Attignon, Marie-Eve Fondrevelle, Arnaud De La Fouchardiere, Maurice Perol, Philippe Cassier, Christelle Seigne, David Perol, Isabelle Ray Coquard, Pierre Meeus, Jerome Fayette, Aude Flechon, Axel Le Cesne, Nicolas Penel, Olivier Tredan, Jean-Yves Blay

**Affiliations:** ^1^ Department of Translational Research, Centre Leon Berard, 69008 Lyon France; ^2^ Department of Medical Oncology, Centre Leon Berard, 69008 Lyon France; ^3^ Department of Clinical Research, Centre Leon Berard, 69008 Lyon France; ^4^ Department of Surgery, Centre Leon Berard, 69008 Lyon France; ^5^ Department of Pathobiology, Centre Leon Berard, 69008 Lyon France; ^6^ Department of Medical Oncology, Centre Oscar Lambret, 59020 Lille France; ^7^ Department of Medical Oncology, Gustave Roussy, 94805, Villejuif France

**Keywords:** multi-kinase inhibitor, biomarker, regorafenib, chromosomal instability

## Abstract

Validated predictive biomarkers for multi-tyrosine kinase inhibitors (MTKI) efficacy are lacking. We hypothesized that interindividual response variability is partially dependent on somatic DNA copy number alterations (SCNAs), particularly those of genes encoding the protein tyrosines targeted by MTKI (called target genes). Genomic alterations were investigated in MTKI responsive and non responsive patients with different histological subtypes included in the ProfiLER protocol (NCT 01774409). From March 2013 to August 2014, 58 patients with advanced cancer treated with one of 7 MTKIs were included in the ProfiLER trial and split into one discovery cohort (*n* = 13), and 2 validation cohorts (*n* = 12 and 33). An analysis of the copy number alterations of kinase-coding genes for each of 7 MTKIs was conducted. A prediction algorithm (SUMSCAN) based on the presence of specific gene gains (Tumor Target Charge, TTC) and losses (Tumor Target Losses, TTL) was conceived and validated in 2 independent validation cohorts. MTKI sensitive tumors present a characteristic SCNA profile including a global gain profile, and specific gains for target genes while MTKI resistant tumors present the opposite. SUMSCAN favorable patients achieved longer progression-free and overall survival. This work shows that the copy number sum of kinase-coding genes enables the prediction of response of cancer patients to MTKI, opening a novel paradigm for the treatment selection of these patients.

## INTRODUCTION

Small molecule antiangiogenic tyrosine kinase inhibitors (TKI), such as regorafenib, sorafenib, sunitinib, pazopanib, axitinib, and cabozantinib, are active in a variety of advanced cancers, including renal cell carcinoma (RCC), gastrointestinal stromal tumors (GIST), hepatocellular carcinoma (HCC), colorectal cancer (CRC) and thyroid cancers [1–9]. These TKIs block multiple membrane-bound and intracellular kinases involved in normal cellular functions which contribute to neoplastic transformation and progression [10, 11]. Compared with single target agents, such as monoclonal antibodies (mAbs), these MTKIs affect multiple enzymes in cancer cells as well as in surrounding cells of the tumor stroma. Predictive criteria for response to these multiple kinase inhibitors (MTKI) are not as well determined as for tumors harboring key driver alterations, such as BCR-ABL translocations in chronic myeloid leukemia (CML), KIT-mutant GIST, BRAF-mutant melanoma, and ALK-positive non-small cell lung cancer among others [5, 9, 12–17]. Regorafenib, for instance, has been shown to yield a progression-free survival (PFS) improvement in pretreated metastatic colorectal cancer (mCRC) and in imatinib and sunitinib refractory gastrointestinal stromal tumors (GIST) [5, 9]. Several approaches to identify biomarkers, such as measuring circulating cytokines related to angiogenesis or drug exposure have been reported, with limited successes to date in predicting response to a given MTKI treatment [18–24]. A general paradigm to identify predictive factors for response to these MTKIs would therefore be of important clinical value.

Most tumors are associated with complex genetic alterations such as gains, losses and point mutations [25–27]. Somatic copy number alterations (SCNAs) are now recognized to play an important role in tumor progression [11]. We hypothesized that response to MTKI may be observed preferentially in cancer cells from patients which have acquired additional copies of genes encoding for the protein targets of the given MTKI. We report here the exploration of this hypothesis in different series of patients treated with MTKIs. We describe an approach that analyses the SCNAs of these kinase-coding genes to predict the response of MTKI therapy across different histological types.

## RESULTS

### Patient characteristics

A total of 58 patients who had received at least one of the 7 MTKIs (listed in Supplementary Table 1) in the first line MTKI setting, were included in the current analysis. Patient characteristics are detailed in Table 1 (more details are given in Supplementary Table 2).

**Table 1:** Patients characteristics

	Discovery cohort	1^st^ Validation cohort	2^nd^ Validation cohort
**Total**	13	12	33
**Age (median, range)**	63.1 (40.7 – 75.8)	56.0 (41.6 – 70.5)	55.9 (24.6 – 76.1)
**Main tumor type n (%)**			
**CRC**^1)^	13 (100%)	7 (58.3%)	3 (9.1%)
**STS**^2)^	0 (0%)	5 (41.7%)	5 (15.2%)
**RCC**^3)^	0 (0%)	0 (0%)	12(36.3%)
**Thyroid**^4)^	0 (0%)	0 (0%)	7 (21.2%)
**HCC**^5)^	0 (0%)	0 (0%)	4 (12.1%)
**MTKIs administered n (%)**			
**Regorafenib**	13 (100%)	12 (100%)	0 (0%)
**Sorafenib**	0 (0%)	0 (0%)	16 (48.4%)
**Sunitinib**	0 (0%)	0 (0%)	12 (36.4%)
**Pazopanib**	0 (0%)	0 (0%)	1 (3.0%)
**Axitinib**	0 (0%)	0 (0%)	1 (3.0%)
**Vandetanib**	0 (0%)	0 (0%)	2 (6.1%)
**Cabozantinib**	0 (0%)	0 (0%)	1 (3.0%)
**Baseline ECOG score n (%)**			
**0**	2 (15.4%)	5 (41.7%)	13 (39.4%)
**1**	4 (30.8%)	3 (25%)	14 (42.4%)
**2**	5 (38.5%)	2 (16.7%)	2 (6.1%)
**NA**^6)^	2 (15.4%)	2 (16.7%)	4 (12.1%)

1) CRC: Colorectal cancer

2) STS: Soft Tissue Sarcoma

3) RCC: Renal Cell Carcinoma

4) Thyroid: thyroid carcinoma

5) HCC: Hepatocellular Carcinoma

6) NA: not available

### Regorafenib treated patients

25 patients received regorafenib as a first-line MTKI. The discovery cohort consisted of 13 patients with metastatic colorectal cancers (mCRC). The median duration of regorafenib treatment was 3 months (range, 0.5 – 25). Six patients who had achieved stable disease (SD) or objective response (partial response, PR or complete response, CR) at 8 weeks were considered as having a clinical regorafenib benefit and 7 patients with progressive disease ≤8 weeks were qualified as having no clinical benefit of regorafenib.

### Establishment of the target copy number change pattern

The copy number alterations of 18 genes encoding for protein kinases targeted by regorafenib were investigated: *RET*, *FLT1* (*VEGFR1*), *KDR* (*VEGFR2*), *FLT4* (*VEGFR3*), *KIT*, *PDGFRα*, *PDGFRβ*, *FGFR1*, *FGFR2*, *TEK* (angiopoietin-1 receptor), *DDR2*, *NTRK1* (High affinity nerve growth factor receptor), *EPHA2*, *RAF1*, *BRAF*, *MAPK11*, *FRK* and *ABL1* [29]. Then, the SCNA profiling of 13 tumors was correlated to clinically defined responses. The sum of gains on target genes was termed as tumor target charge (TTC), while the sum of losses of target genes were termed as tumor target loss (TTL). Each type of gain events (amplification, gain & heterogeneous gain) were considered as 1 TTC and each loss event (gene loss, deletion, heterogeneous deletion) were considered as 1 TTL.

**Figure 1:**
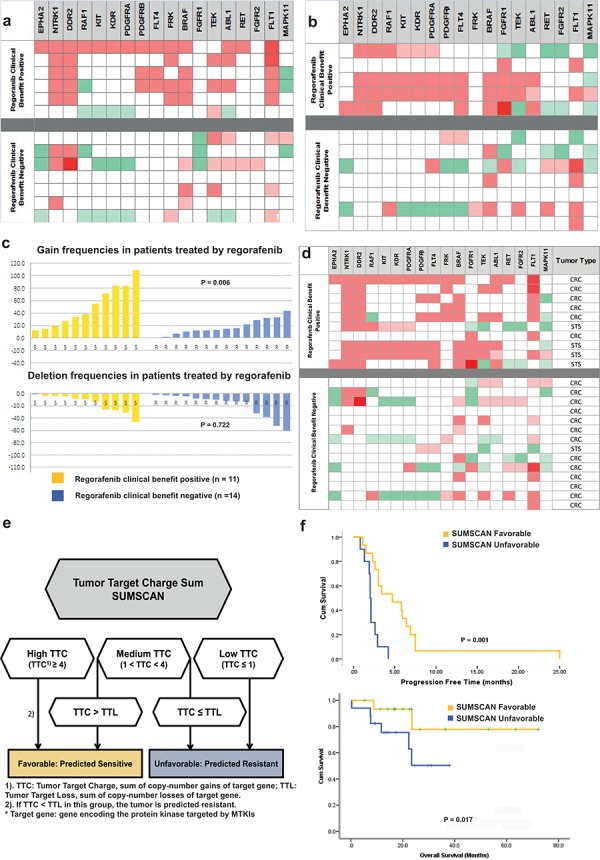
**a.** Copy-number alteration pattern of the 18 regorafenib target genes in the discovery cohor. The SCNA pattern of the 18 target genes was displayed as a heatmap. Top and bottom parts show the grouped results of the 6 regorafenib clinical benefit positive and the 7 regorafenib resistant tumors, respectively. The tumor/normal log_2_ ratios categories for different copy-number alterations levels were defined as in the online methods. **b.** Copy-number alteration pattern of the 18 regorafenib target genes in the validation cohort I. Top and bottom parts show the grouped results of the 5 tumors with regorafenib clinical benefit and the 7 tumors without clinical regorafenib benefits, respectively. **c.** Sum of total gains and deletions in the regorafenib clinical benefit positive and the clinical benefit negative tumors. There are significantly more gene gains in tumors having a clinical benefit than in the resistant ones (Top). **d.** Integral analysis of copy-number change pattern of the 18 regorafenib target genes in 25 patients. Gene gain events in red and gene deletion events in green. **e.** SUMSCAN Algorithm 1). TTC: tumor target charge, sum of gains on target genes; 2). Number of gains at target genes* versus number of losses at target genes 3) If there are more losses at target genes than gains, the tumor is predicted as resistant. All gain/loss events were equally considered. **f.** PFS and OS curve of 25 patients treated with regorafenib.

Enrichment in gains of genes encoding for regorafenib targets was observed in tumors from patients having a clinical benefit with a total of 41 gains (mean: 6.8; range 1–14) versus 20 gains for tumors from patients without clinical benefit (mean: 2.1; range 0–7) (Fig. 1a). Furthermore, tumors from patients with clinical benefit had a total of 8 losses (mean: 1.3; range 0–5), while the tumors from patients without clinical benefits had a total of 17 losses (mean: 2.4; range 0–7). The differences between TTC and TTL were significantly higher in the group with clinical benefit compared to the group without clinical benefit (*p* = 0.038; Mann Whitney). Additionally, five out of six tumors with clinical benefit had a TTC ≥ 4, versus 2 of 7 tumors with no clinical benefit (*p* = 0.048). The details of SCNAs of all patients are listed in the Supplementary Table 4.

In the regorafenib validation cohort, a high TTC was observed only in the tumors with clinical benefit (Fig. 1b). The differences between TTC and TTL was significantly higher in these tumors (Mann Whitney, *p* = 0.014). Four of these tumors had a TTC ≥ 4, versus two of seven for tumors with no clinical benefit (*p* = 0.07; Fisher's Exact Test) (Fig. 1b).

The pooled analysis of the 2 regorafenib sets (Fig. 1d) revealed that the TTL tends to outnumber TTC in tumors without clinical benefit. As expected, the difference between TTC and TTL were significantly higher in the tumors with clinical benefit (*p* = 0.003; Mann Whitney). Of note, all 8 patients with a large difference (definition: TTC – TTL ≥ 5) achieved clinical benefit, vs 3 of the 17 remaining patients (*p* = 0.0001, Fisher's Exact Test). The analysis of the subgroup of patients with mCRC treated with regorafenib who had PFS ≥4 months revealed an average TTC of 7 and TTL of 0.5. Combining the two parameters enabled to define an algorithm, termed SUMSCAN (Fig. 1e). Using this algorithm, the patients were split into favorable and unfavorable groups, which may translate into a prediction for clinical benefit or no in the clinical context. Ten of the eleven clinical benefit tumors would have been identified as favorable, versus five of the fourteen remaining patients, resulting in a sensitivity of 90.9% and a specificity of 66.7%. A prediction accuracy of 76% (19 of 25) was achieved. Furthermore, the prognostic significance of SUMSCAN was evaluated. Patients with a favorable SUMSCAN profile achieved better PFS and OS ( *p*_PFS_ = 0.001, *p*_OS_ = 0.017, respectively; Log-rank test, Fig. 1f). The total gain events or TTC were not a predictive marker for PFS or OS benefits (see Supplementary Figure. 3).

### Gains and deletions for specific target genes

**Figure 2:**
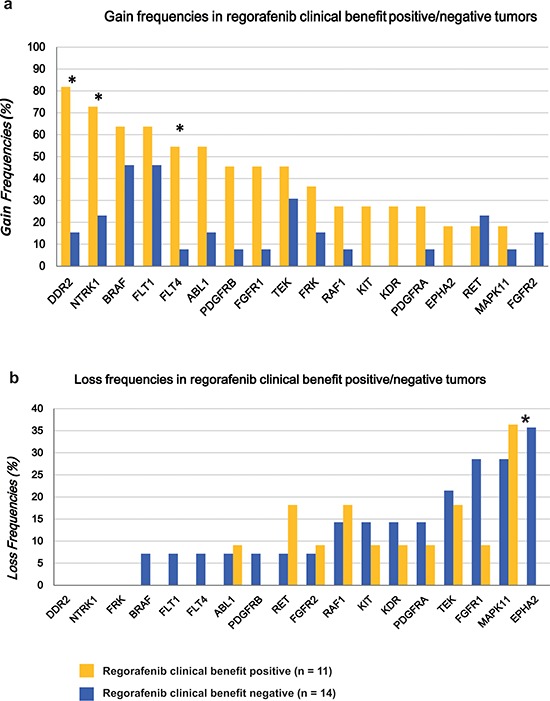
Differences concerning gain and loss frequencies between patients with regorafenib clinical benefits and those without clinical benefits **a.** A significant difference in the gain frequencies was observed for the *DDR2*, *NTRK1* and *FLT4* genes. **b**. Specific loss on EPHA2 was remarkably frequent in the regorafenib resistant tumors.

A significant difference in the gain frequencies between regorafenib clinical benefit positive tumors and regorafenib clinical benefit negative tumors was observed for *DDR2*, *NTRK1* and *FLT4* genes (Fig. 2a). Gains of *DDR2*, *NTRK1* and *FLT4* were observed in 81.8% (9/11), 72.7% (8/11) and 54.5% (6/11) of 11 tumors having clinical benefit but only in 14.3% (2/14), 21.4% (3/14) and 7.1% (1/14) of the tumors without clinical benefit (*p* < 0.05 each, Fisher exact test). Additionally, specific losses of *EPHA2* were observed in 42.9% (6/14) of the tumors without clinical benefit but in none of the tumors having clinical benefit (Fig. 2b). No association was observed for the other 12 genes analyzed. Even though several of these 18 individual gene gains helped to predict regorafenib clinical benefit, the numerical combination of TTC and TTL appeared to be the most efficient predictor of clinical benefit for regorafenib treatment.

### Overall gains in the genome of sensitive vs resistant patients

As a control, we compared global genome gains and losses in the same experiment (Fig. 1c). A total of 545 gains across 187 genes were observed in the clinical benefit positive group (mean: 49.5; range 12–109). In contrast, a total of 246 gains across 187 genes was observed (mean: 16.7; range 0–44) (*p* =0.006; Mann-Whitney) showing that patients with clinical benefit present an overall “gain” profile. Regarding the gene losses, a total of 175 losses in the clinical benefit positive group (mean = 15.9, range 1–46) versus 265 losses (mean = 18.9, range 2–61) in the resistant group were observed (*p* = 0.722; chi-square). The sum of copy-number gains minus copy-number losses of the genes not encoding targets of regorafenib were not significantly higher in tumors from the group of patients with clinical benefit (*p* = 0.17, Mann Whitney). The genomic index was not significantly different between the two groups (Data not shown).

### Added predictive ability of mutation analysis for the same samples using NGS

We then investigated the correlation between mutations in 59 cancer related genes (Genes list as Supplementary Table 3) and response to regorafenib, with the aim of improving the predictive value of SUMSCAN. *PIK3CA* mutation (42.9% in resistant tumors vs none of the tumors having clinical benefits, *p* = 0.0196; Fisher exact test) was found associated with resistance, as opposed to *TP53* (72.7% in clinical benefit tumor vs 64.3% in no clinical benefit tumors) and *KRAS* (27.3% in sensitive tumor vs 57.1% in resistant tumors). *PIK3CA* mutations were mainly located in exon 9 (E542K*2, E545K*2, Q546P) and one in exon 20 (H1047R), five patients with PIK3CA-mutant tumors are in the low TTC (TTC ≤ 2) sub-group, and 4 of 6 were classified unfavorable by SUMSCAN. This association was only observed in mCRC.

### Second validation series: assessment of SUMSCAN in tumors treated by other MTKIs

**Figure 3:**
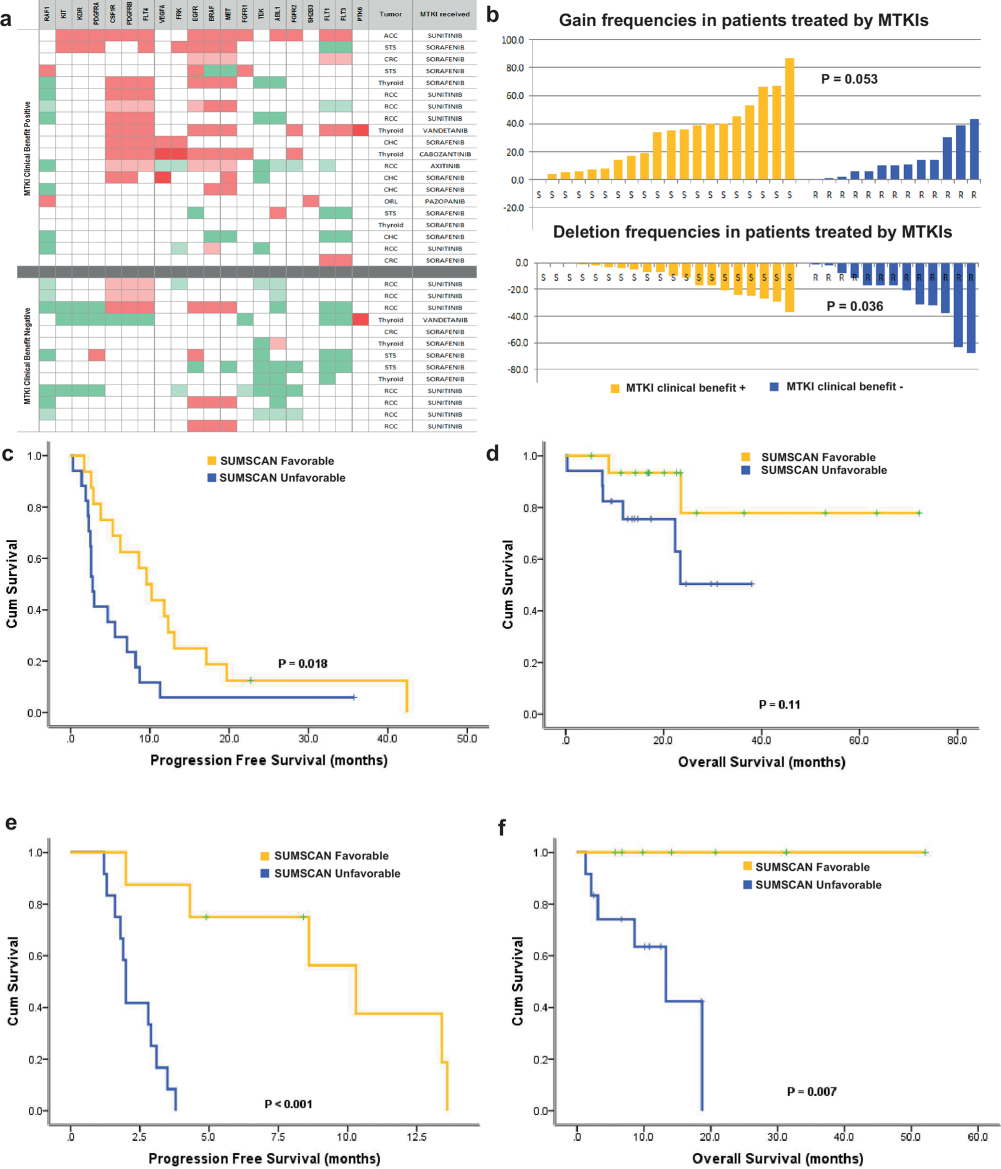
Validation II in 33 patients treated by one of the 6 other MTKIs **a.** Copy-number alteration pattern of the target genes of 6 MTKIs (Sorafenib, sunitinib, pazopanib, axitinib, vandetanib and cabozantinib) in the validation cohort: Top and bottom parts show the results of the 20 tumors with clinical benefits and the 13 tumors without clinical benefits, respectively. **b.** Sum of total gains and deletions in the validation cohort II. There are significantly less gene deletions and more gene gains in the tumors having the clinical benefits compared to those without clinical benefits. **c.** and **d.** PFS and OS curve of 33 patients treated with one of the 6 MTKIs in the first-line MTKI settings. The patients with a favorable SUMSCAN had a progression free survival benefit. **e.** and **f.** PFS and OS curve of 22 patients treated by MTKI in the second-line MTKI settings. Patients with a favorable SUMSCAN achieved a better PFS and OS.

The predictive power of SUMSCAN was further assessed in 33 patients treated with 6 other MTKIs (Fig. 3a). The details of SCNAs and mutation of each target gene were listed (Supplement Table 5). The definition of target genes varied between each MTKI according to DrugBank [29]. Again, the difference between TTC and TTL was significantly different between clinical benefit positive (*n* = 20) and clinical benefit negative tumors (*n* = 13) (*p* = 0.008; Mann-Whitney). Tumors from fourteen of twenty patients with clinical benefits were classified as “favorable” by SUMSCAN, versus two of the thirteen resistant patients (*p* = 0.002; Fisher's Exact Test). In the validation set, SUMSCAN succeeded in obtaining a sensitivity of 70% and a predictive accuracy of 75.8%. The positive predictive value (PPV) and negative predictive value (NPV) were 87.5% and 64.7%, respectively. Patients with a favorable SUMSCAN profile had a median PFS of 9.9 months versus 2.8 months for SUMSCAN unfavorable patients, and a trend for a better overall survival (Fig. 3c and Fig. 3d).

In this validation cohort, we found a total of 622 gains in clinical benefit positive tumors (mean: 31.1; range 0–87) versus 186 gains in progressive patients (mean:14.3; range 0–43) (*p* = 0.053, Mann-Whitney), and 247 losses in 20 samples of clinical benefit positive patients (mean: 12.4; range 0–37) versus 327 losses in the samples of resistant patients (mean: 25.2; range 1–68) (*p* = 0.036; Mann-Whitney) (Fig. 3b). The genomic index is not significantly different between two groups. The total gain events or TTC were not a predictive marker for PFS or OS benefits, either (Fig. S4).

### Second line and beyond

We then analyzed the utility of SUMSCAN in 22 patients treated with 2^nd^–or-further line of MTKIs. Three of twenty-two patients were treated by more than 2 MKTIs. This analysis was conducted in 26 cases (one case = one patient treated by one MTKI). Twenty-two second-line cases were included in survival analysis (Fig. 3e and 3f). SUMSCAN predicted 10 out of 12 patients with clinical benefit, and 12 or 14 patients who derived no befinit from 2nd line treatment, with an accuracy rate of 84.6%, a sensitivity of 83.3% and a specificity of 85.7% (*p* = 0.0011, Fisher's Exact test). Interestingly, two patients had a TTC superior to that of the 1^st^ line, both had experienced a longer PFS than that in the 1^st^ line setting, one with thyroid carcinoma had a PFS of 19 months with vandetanib (TTC = 3) and 35 months with sunitinib (TTC = 6) in the 2^nd^ line. Similarly, patients with a favorable SUMSCAN had significantly better PFS and OS (p_PFS_ < 0.001, p_OS_ = 0.007, Fig. 3e and Fig. 3f).

### SUMSCAN is not predictive of the response to conventional chemotherapy

To evaluate the specificity of SUMSCAN on the prediction of MTKI outcomes, the correlation between SUMSCAN and response to conventional chemotherapy regimens (irinotecan and oxaliplatin containing regimens) as well as response to epidermal growth factor receptor (EGFR) inhibitors containing regimen (data not shown) received beforehand was evaluated in 21 mCRC patients (see Table 2). No correlation was observed.

**Table 2:** SUMSCAN score and response to chemotherapy in mCRC patients (*n* = 21)

Irinotecan Sensitivity	SUMSCAN Favorable	SUMSCAN Unfavorable^2)^	Total
Sensitive	9 (42.9%)	7 (33.3%)	16 (76.2%)
Resistant	3 (14.3%)	2 (9.5%)	5 (23.8%)
***P* = 1 (Fisher Test)**			
**Oxaliplatin Sensitivity**	**Predicted Sensitive**	**Predicted Resistant**	**Total**
Sensitive	6 (28.6%)	7 (33.3%)	13 (61.9%)
Resistant	6 (28.6%)	2 (9.5%)	8 (38.1%)
***P* = 0.366 (Fisher Test)**			

All patients have been treated by both irinotecan-based regimen and oxaliplatin-based regimen before regorafenib. Nine patients with a SUMSCAN Favorable were judged sensitive to irinotecan-based chemotherapy and 3 other patients were judged resistant to irinotecan-based regimen

## DISCUSSION

In this study, we report that the antitumor activity of MTKIs in tumors lacking a well-defined oncogenic driver is strongly correlated with copy number alterations of genes encoding the protein kinases targeted by these drugs. A concept of tumor target charge (TTC), defined as the total gains of the genes encoding for targets of MTKIs as well as tumor target loss (TTL) was developed, and correlated to response to regorafenib in 2 cohorts of patients composed of mCRC and STS patients. A predictive model, called SUMSCAN, was conceived as a binary classifier to identify patients as either good or poor candidates for use of MTKIs. Moreover, the PFS and OS of patients with a favorable SUMSCAN score were significantly improved. Importantly, SUMSCAN predicted exclusively response to regorafenib, but not the response to conventional chemotherapy in mCRC.

We then tested the generalizability of SUMSCAN in tumor types treated with MTKIs. A variety of histological subtypes and six different MTKIs (sorafenib, sunitinib, pazopanib, axitinib, vandetanib and cabozantinib) were included in the 2^nd^ validation cohort. Again, TTC was higher in patients with clinical benefit across various histological types and MTKIs. In the 2^nd^ line setting, patients with a favorable SUMSCAN achieved a better PFS (median PFS = 7 months) than those with an unfavorable profile (median PFS = 2.4 months). It seems that SUMSCAN may be a transversal marker predicting clinical outcome in a broad array of cancers having indications of MTKI treatment, suggesting that SUMSCAN reflects a fundamental biological characteristics of these tumors. (Supplementary Figure 1 & 2).

SCNAs of individual genes encoding MTKI targets were found to be predictive of clinical benefit, but taken individually, none were as precise overall as the numerical combination of TTC and TTL described in SUMSCAN. Furthermore, we analyzed the mutation status of a panel of cancer-related genes: only *PIK3CA* mutations were exclusively observed in regorafenib resistant mCRC tumors with low TTC. No other mutations were associated with regorafenib resistance, including *KRAS Tp53*/BRAF mutations. This suggested that *PIK3CA* mutations could further refine the SUMSCAN prediction for regorafenib resistance in mCRC. The mutually exclusive presence of *PIK3CA* mutations and high TTC observed in this study is consistent with the cancer genome hyperbola which describes the fact that tumors at the extremes of genomic instability had either a large number of somatic mutations or a large number of copy number alterations, never both [30].

Another important observation is that tumors from patients showing clinical benefit present a globally high frequency of copy-number gain and low frequency of copy-number loss profile, while the resistant tumors are the opposite. This phenomenon was not observed in oxaliplatin/irinotecan sensitive/resistant mCRC (data not shown). Patients with tumors presenting a “gain profile” may therefore be candidates for therapies targeting multiple oncogenes.

These results also challenge the antiangiogenic role of these MTKI as a major component of their antitumor activity. Indeed, while prolonged clinical benefit was observed in some patients whose tumors do not present mutations of oncogenes, the analysis of the SUMSCAN score and TTC of individual tumors suggest that the antitumor activity of these agents is exerted primarily on the tumor cells which gained additional copies of genes encoding for MTKI targets. This question is also important for patients with KIT-mutant GIST treated with regorafenib or sunitinib. We are currently exploring this question in a large dataset of patients treated in 2^nd^ or greater line with these MTKIs. It is important to note that the antitumor activity of regorafenib was observed at the same level regardless of the nature of the KIT/or PDGFRA mutation [9].

There are potential limitations in this study that need to be mentioned. Firstly, the prediction model does not integrate the level of target gene gains. Whether the existence of target gene amplification will reinforce sensitivity or become a predominant target remains to be further investigated. Secondly, given the relatively small sample size and heterogeneity of patients, it is essential that these results should be replicated in additional independent data cohorts. A follow-up study shall be conducted when additional data from the ongoing profiLER study become available.

In conclusion, these results point to a novel concept that the response to any MTKI in human solid tumors is influenced by the somatic copy number alteration of the genes encoding the protein targets of this MTKI in the tumor. A predictive model for the selection of patients is presented and proposed for future evaluation in other series. These results could have important consequences for a better selection of candidates for these treatments in the routine clinical setting. In addition, these results may be useful in the identification of patients that may benefit from these MTKIs regardless of their tumor type, enabling registration of an already approved agent in additional indications. Finally, the concept that the sum of gains and losses of genes encoding target proteins is predictive for treatment efficacy may have broader application beyond MTKI targeting VEGFRs: for example the identification of patients sensitive to multitargeted inhibitors of ALK, MET, SRC, mTOR/PI3KCA/AKT, for instance, may be worth exploring with this concept, consistently with the observation that multiple gene alterations are critical for the progression to malignancy [11]

## MATERIALS AND METHODS

### Study design and patients

Patients included in the profiLER prospective program (NCT01774409) treated with MTKI in advanced stages of disease were included. The profiLER study aims to establish a genetic profile of advanced tumors by CGH and targeted mutation sequencing. As of Nov 2014, 1163 patients have been included.

### Patients treated with regorafenib (*n* = 25)

Among the first 700 patients enrolled in the program from March 2013 to March 2014, 23 patients with pretreated mCRC and 5 patients with advanced STS received regorafenib from February 2011 to February 2014 (Table 1 and Supplementary Table 3) under the ATU, a compassionate use program of the French National Agency of Medicine and Health Products Safety. Twenty-five tumor samples fulfilled DNA quality requirements for analysis. These patients were split into a discovery cohort of 13 mCRC patients and the first validation cohort of 12 patients, with 7 mCRC and 5 STS patients. The patients received regorafenib at standard doses of 160 mg or 120 mg daily as first-line MTKI, with dose de-escalation according to standard recommendations.

### Patients treated with other MTKI (*n* = 33)

The second validation cohort consisted of 33 profiLER patients treated with one of the 6 following MTKIs: sorafenib, sunitinib, pazopanib, axitinib, vandetanib and cabozantinib. The predictive model was additionally tested in a subset of 22 of these 33 patients who have received further MTKI.

### Outcomes

Patients included in this analysis had CT examinations of the thorax, abdomen and pelvis performed at the center, 4 +/− 2 weeks before and 8 +/− 2 weeks after initiation of MTKI treatment. Baseline demographic and clinical data were collected, with the site and dates of metastases, previous systemic therapies, MTKI treatment, treatment duration, and response and progression free survival (PFS) determined by the radiologist and physician at each follow-up visit with the Response Evaluation Criteria in Solid Tumors (RECIST, version 1.1) [28]. Best response and the PFS (defined as the time elapsed between treatment initiation and first tumor progression or death) were collected. Patients with complete response, partial response and stable disease lasting at least 2 months were defined as “MTKI clinical benefit positive”; those with progressive disease as best response at or before 8 weeks were classified as “MTKI clinical benefit negative”.

### Tumor sampling and DNA extraction

All the tumor samples were collected before MTKI treatments. Forty samples were collected from the primary tumor (69.0%) and eighteen from metastasis (31.0%), all from paraffin embedded tissues. Small amounts of tumors were then collected by directly scraping the blocks in the most representative areas. Genomic DNAs were then extracted using the Deparaffinization Solution (Qiagen #19093) and the QIAamp DNA Micro Kit (Qiagen #56304). DNAs were eluted in 20 μl of DNAse-free water.

### SCNAs analysis with array CGH

Fragmentation and labeling were carried out according to the manufacturer's recommendations for the CGH array (Agilent Technologies, Santa Clara, CA). In brief, 1.5 μg of tumor DNA and 1.5 μg of reference DNA (Promega #G1471 or #G1521, WI, USA) were heat denatured and fragmented for 10 min at 95°C. Then, tumor DNA was chemically labeled with Kreatech's Universal Linkage System (ULS™) Cy5-dye, whereas reference DNA was labeled with Cy3-dye (Agilent #5190-0450). Labeled samples were then purified using KREApure columns (Agilent #5190-0418). Co-hybridization was performed on 4*180K Agilent SurePrint G3 Human whole-genome oligonucleotide arrays (Agilent #G4449A), containing 180 000 oligonucleotide probes. Slides were washed, dried and scanned on the Agilent Surescan scanner according to the manufacturer's recommendations. Scanned images were processed using Agilent Feature Extraction software V11.0 and analyses were carried out using the Agilent Genomic Workbench software V7.0. The identification of aberrant copy number segments was based on ADM-2 segmentation algorithm with a threshold of 7.0. A null Log_2_ ratio corresponds to a balanced tumor/normal DNA ratio. Low-level and high-level copy number gains/losses were defined as a |log_2_ (ratio)| > 0.25 and 1.5, respectively. Gene Amplification: log_2_ ratio 2; Strong Gain: 1 ≤ log_2_ ratio < 2; Gain: 0.5 ≤ log_2_ ratio < 1; Heterogeneous Gain: 0.1 ≤ log_2_ ratio < 0.5; Gene Loss: log_2_ ratio ≤ −1; Deletion: −1 < log_2_ ratio ≤ −0.5; Heterogeneous Deletion: −0.5 < log2 ratio ≤ −0.1. The genomic index was calculated for each profile as follows: Genomic Index = A2/C, where A is the total number of alteration (segmental gains and losses) and C is the number of involved chromosomes.

### Somatic mutation detection with NGS

Twenty nanograms of tumor DNA were used for the Ion Torrent library preparation of a panel covering 59 actionable genes (Supplementary Table 3) following the manufacturer's protocol for the Ion AmpliSeq Library Kit 2.0 (Life Technologies #4475345). The size distribution of the DNA amplicons was analyzed on the 2200 TapeStation (Agilent) using the High sensitivity kit (Agilent #5067-4626). Template preparation, emulsion PCR, and Ion Sphere Particle (ISP) enrichment was performed using the One Touch 2 kit (Life Technologies) according to manufacturer's instructions. The ISPs were loaded onto a 318 chip (Life Technologies #4484355) and sequenced using an Ion PGM 200 V2 sequencing kit (Life Technologies #4482006) on the Ion Torrent PGM for 500 cycles. The raw signal data were analyzed using NextGENe Software Suite v3.4.2 (Soft genetics).

The pipeline includes quality score assignment, alignment to the human genome 19 reference, mapping quality QC, coverage analysis and variant calling. After completion of the primary data analysis, lists of detected sequence variants (SNVs and INDELs) were compiled in the VCF (Variant Call File) format. For downstream analysis, variants with minimum coverage of 100 reads containing at least 10 of the mutant reads were selected. Variant calls were further analyzed using variant filtering and annotation using COSMIC v.64 and dbSNP build 135.

### Statistical analysis

The association between SCNAs and categorical variables was tested using the Mann-Whitney *U*-test. Association between categorical variables was assessed using Chi-square test. All *p*-values were two-sided. Survival curves were plotted using the Kaplan-Meier method and compared using a log rank test. Statistical analysis was conducted using the SPSS 19.1 Package (SPSS, IBM France).

## SUPPLEMENTARY FIGURES, TABLES LEGENDS


