# Supplementary material for: The sum of gains and losses of genes encoding the protein tyrosine kinase targets predicts response to multi-kinase inhibitor treatment: Characterization, validation, and prognostic value
Source: Oncotarget. 2015 Jul 21;6(28):26388–99. doi: 10.18632/oncotarget.4557 (PMC4694909; doi:10.18632/oncotarget.4557)
Supplement: Supplementary file 1 [file oncotarget-06-26388-s001.pdf]

SUPPLEMENTARY FIGURES, TABLES LEGENDS

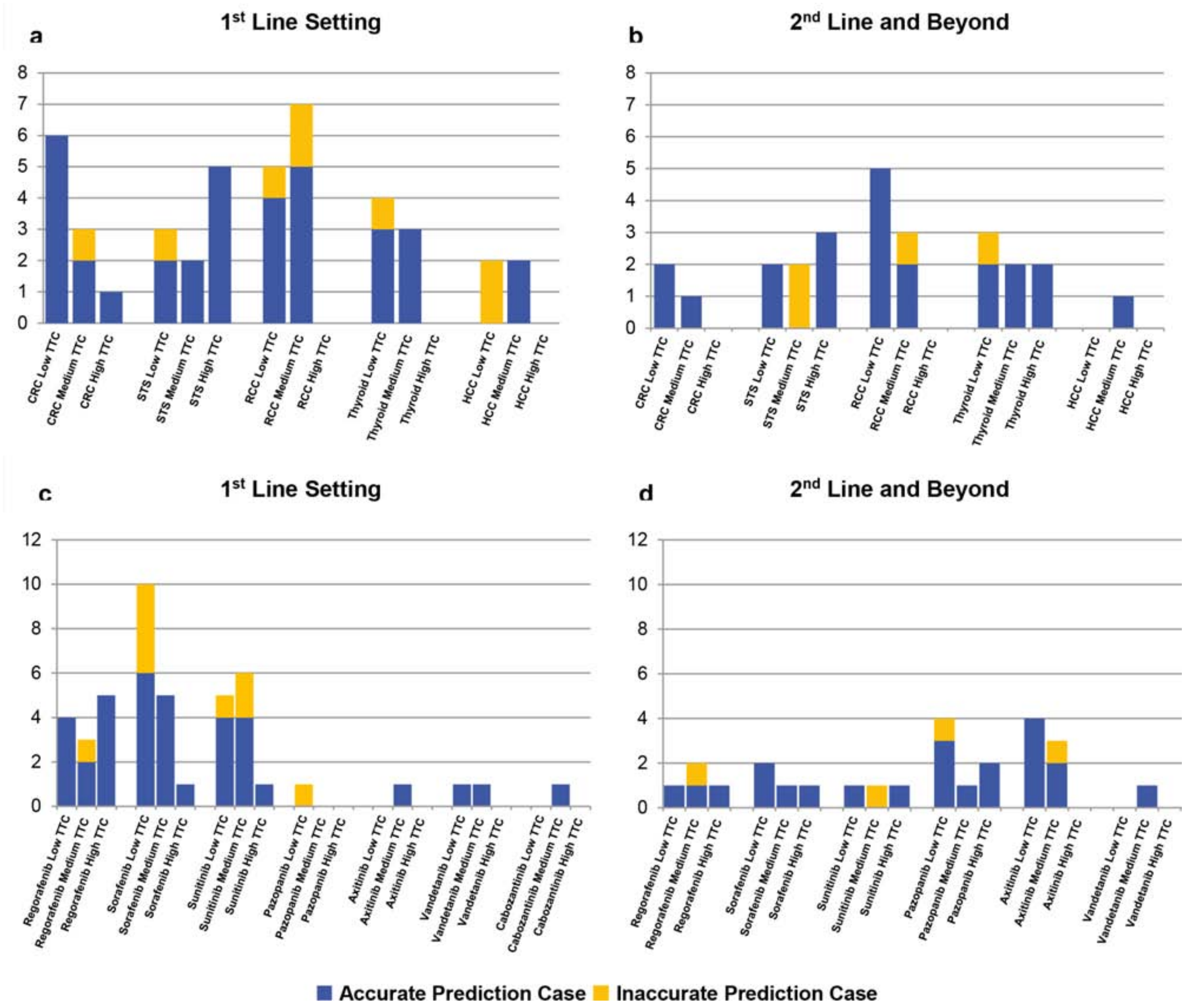

Supplementary Figure S1: a. SUMSCAN performance in 5 histological types treated by MTKI in the 1st line. Each histological subgroup is divided into 3 groups according to TTC (Low TTC ≤ 1; Medium TTC = 2, 3; High TTC ≥ 4) from left to right. The blue bar shows the total number of patients for whom SUMSCAN succeed to predict the clinical outcome. The yellow bars show the number of patients for whom SUMSCAN failed to predict the clinical outcome. **b.** SUMSCAN performance in 5 histological types treated by MTKIs in the 2nd line. **c.** SUMSCAN performance in 7 MTKIs applied as 1st line MTKI treatment. **d.** SUMSCAN performance in 7 MTKIs applied as 2nd line MTKI treatment. As shown in 4 figures, no discordant cases observed in high TTC subgroup across different histological types and different MTKI in all line setting.

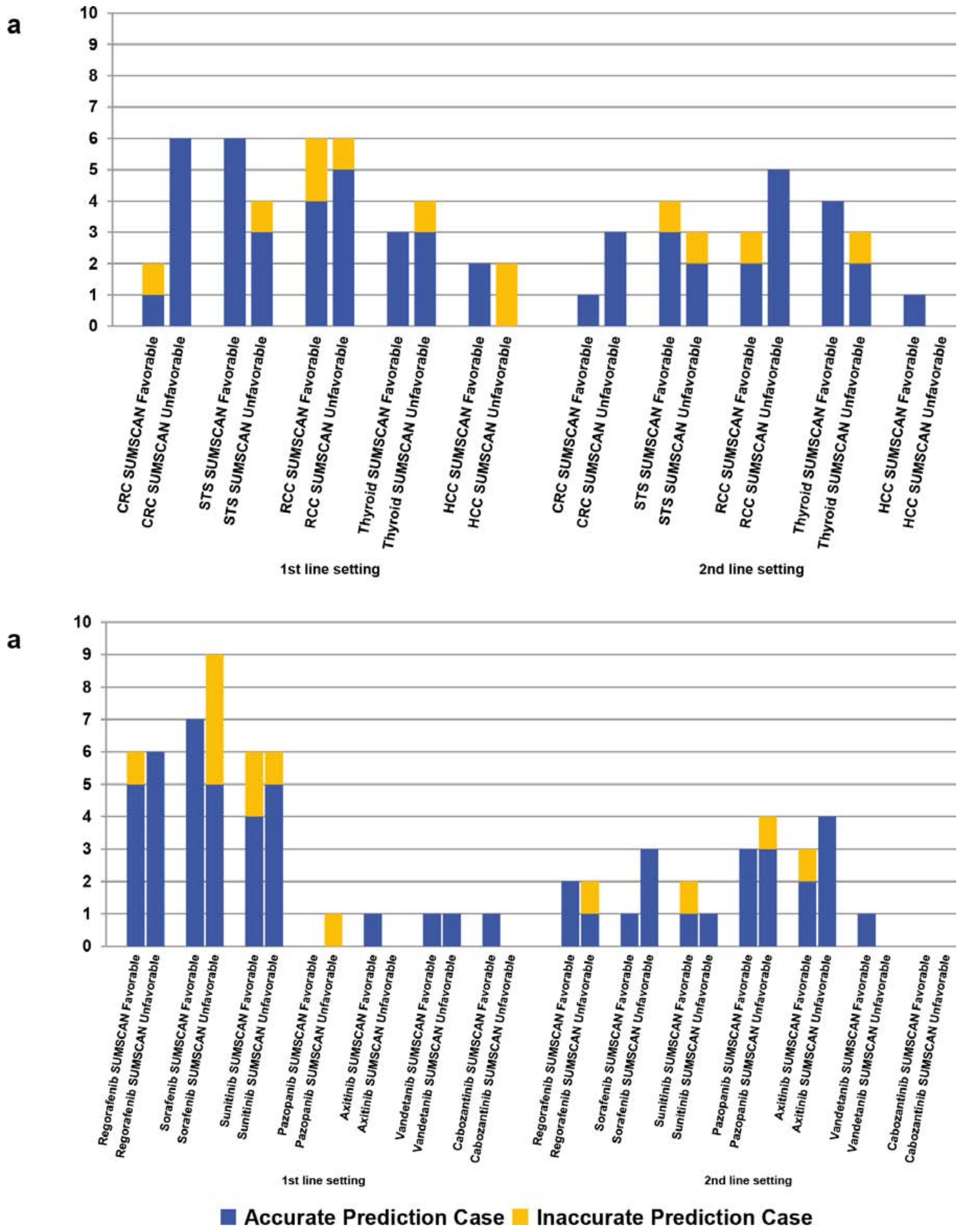

Supplementary Figure S2: a. Prediction Model Accuracy in Different Tumor Types and MTKIs (SUMSCAN Classification). Each histological subgroup is divided into 2 groups according to SUMSCAN (Favorable vs Unfavorable) from left to right CRC, STS, RCC, Thyroid carcinoma and HCC. The blue bar shows the number of patients for whom SUMSCAN succeed to predict the clinical outcome. The yellow bars show the total number of patients for whom SUMSCAN failed to predict the clinical outcome. **b.** SUMSCAN performance across 7 MTKIs used in the 1st line and 2nd line. From left to right: regorafenib, sorafenib, sunitinib, pazopanib, axitinib, vandetanib and cabozantinib.

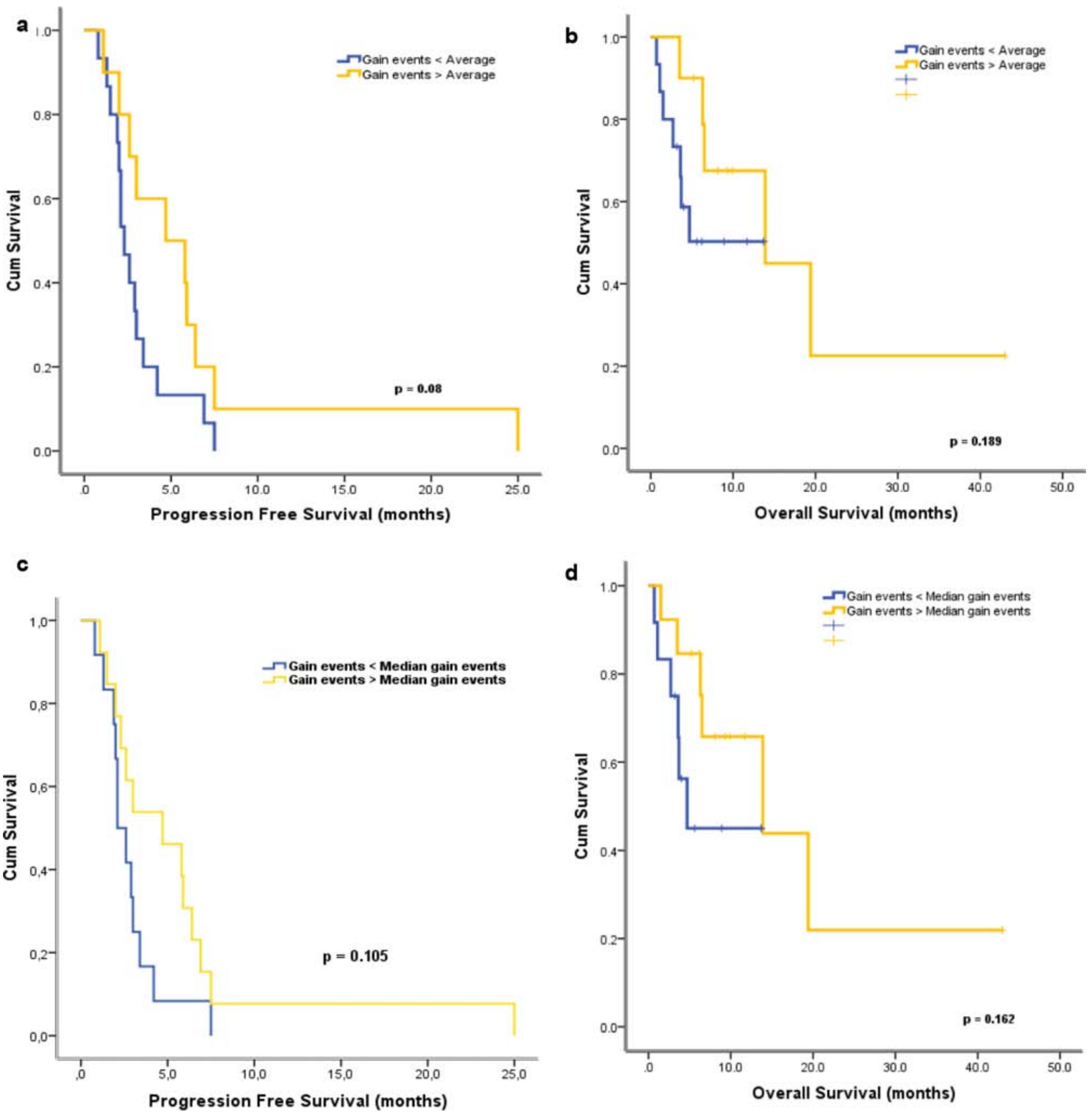

Supplementary Figure S3: Survival curves in regorafenib cohort . 25 patients treated with regorafenib as 1st line MTKI were divided into two groups according to their total gain events (Yellow: total gain events above the average; blue: total gain events below the average). No differences were observed between two groups for PFS and OS curves. **c** and **d.** 25 patients treated with regorafenib as 1st line MTKI were divided into two groups according to their total gain events (Yellow: total gain events above the median; blue: total gain events below the median). No differences were observed between two groups for PFS and OS curves.

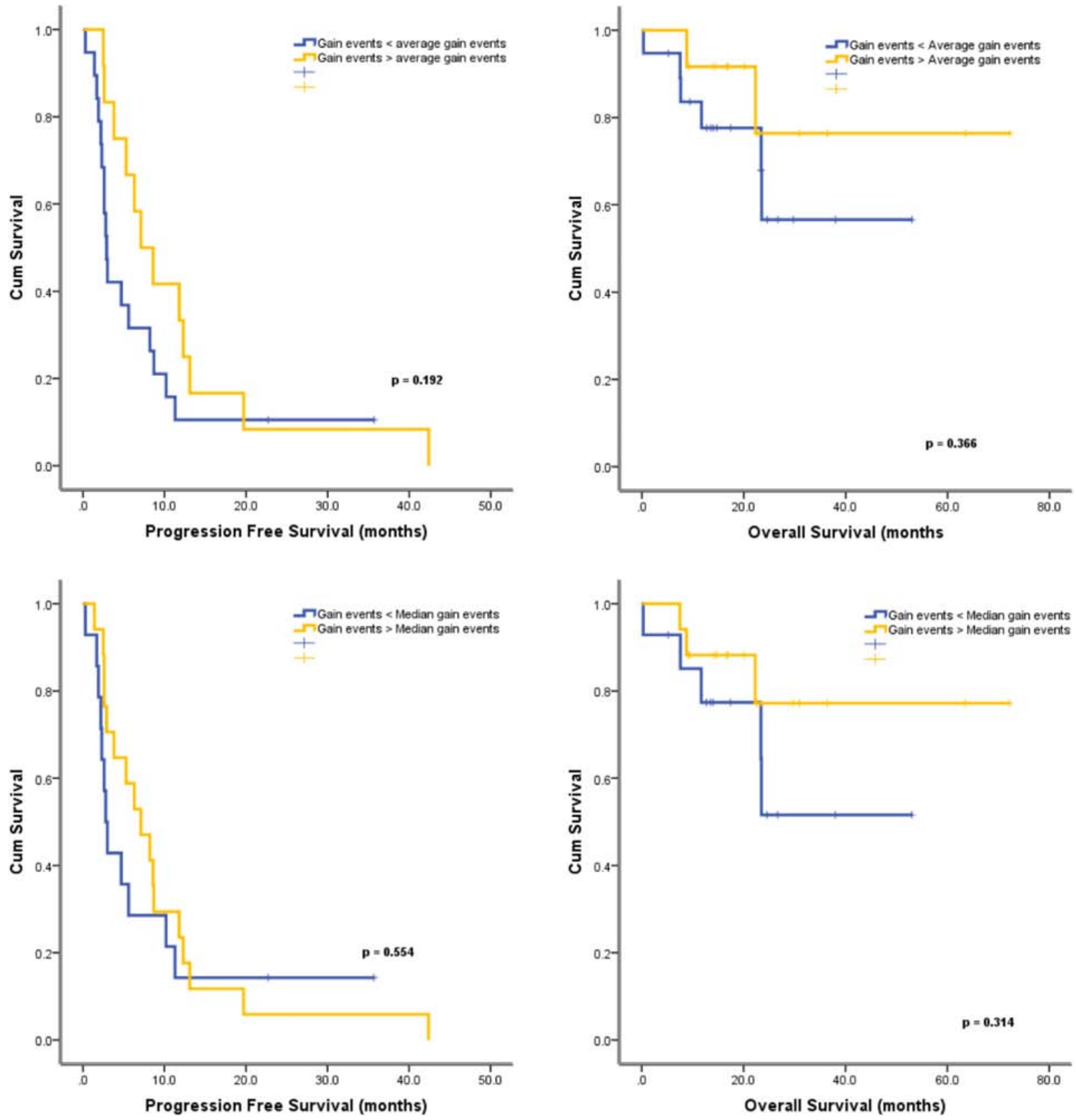

Supplementary Figure S4: Survival curves in MTKI cohort. 33 patients treated with other 6 MTKIs (sorafenib, sunitinib, pazopanib, axitinib, vandetanib & cabozantinib) as 1st line MTKI were divided into two groups according to their total gain events (Yellow: total gain events above the average; blue: total gain events below the average). No differences were observed between two groups for PFS and OS curves. **c** and **d.** 33 patients treated with 6 other MTKIs as 1st line MTKI were divided into two groups according to their total gain events (Yellow: total gain events above the median; blue: total gain events below the median). No differences were observed between two groups for PFS and OS curves.

Supplementary Table S1: Patients characteristics

N°	Gender	Age	Tumor Type	Time to metastasis (month)	MTKI received	Number of previous lines of chemotherapy
**Discovery Cohort**						
1 BR	M	64.5	CRC[1)]	14.1	Regorafenib	2
2 LJ	F	61.5	CRC	7.6	Regorafenib	5
3 CB	F	65.4	CRC	25.1	Regorafenib	10
4 DH	M	72.2	CRC	12.0	Regorafenib	5
5 CP	M	69.0	CRC	0	Regorafenib	4
6 BZ	F	53.2	CRC	34.3	Regorafenib	4
7 JJ	F	60.2	CRC	0	Regorafenib	2
8 BM	M	69.7	CRC	0	Regorafenib	5
9 MB	M	72.7	CRC	0	Regorafenib	5
10 RS	F	40.7	CRC	0	Regorafenib	5
11 AM	M	75.8	CRC	11.0	Regorafenib	3
12 MC	M	65.4	CRC	0	Regorafenib	2
13 LH*	M	51.2	CRC	1.9	Regorafenib	4
**1st Validation Cohort**						
1 PN	F	55.1	CRC	0	Regorafenib	> 2
2 AS	F	41.6	STS[2)]	23.1	Regorafenib	2
3 PA	M	68.9	STS	4.4	Regorafenib	1
4 GN	F	57.3	STS	0	Regorafenib	2
5 GE	F	67.3	STS	5.3	Regorafenib	3
6 CJC	M	53.4	CRC	0	Regorafenib	6
7 PE	F	42.8	CRC	NA[3)]	Regorafenib	3
8 PN	F	58.6	CRC	0	Regorafenib	2
9 NA	F	70.5	CRC	28	Regorafenib	4
10 CF	F	67.8	CRC	0	Regorafenib	3
11 BE	M	44.1	STS	1.3	Regorafenib	2
12 RD	M	44.4	CRC	0	Regorafenib	2
**2nd Validation Cohort**						
1 VOM	F	53.9	ACC[4)] Carcinoma	0	Sunitinib	2
2 NL*	F	76.1	STS	NA	Sorafenib	2
3 DE	M	45.3	CRC	0	Sorafenib	6
4 DBO*	F	70.7	SRC	0	Sorafenib	3
5 MC*	M	47.1	Thyroid[5)]	4.1	Sorafenib	0

(*Continued*)

N°	Gender	Age	Tumor Type	Time to metastasis (month)	MTKI received	Number of previous lines of chemotherapy
6 CC	F	49.8	RCC[6)]	86.4	Sunitinib	0
7 DP*	M	53.4	RCC	20.4	Sunitinib	0
8 GAM*	M	69.1	RCC	23.8	Sunitinib	1
9 PJ**	M	54.4	Thyroid	44.2	Vandetanib	0
10 TM	F	65.6	HCC[7)]	5.0	Sorafenib	0
11 BR*	M	59.0	Thyroid	24.7	Cabozantinib	0
12 BJM	M	59.8	RCC	2	Axitinib	0
13 GS	M	64.9	CHC	19.9	Sorafenib	0
14 PF*	F	62.9	HCC	6.5	Sorafenib	0
15 CC	F	54	Head&Neck	0	Pazopanib	3
16 JD*	M	55.1	STS	21.2	Sorafenib	3
17 DG*	M	68.2	Thyroid	0	Sorafenib	0
18 AJ	F	50.4	HCC	4.5	Sorafenib	1
19 RJ*	M	65.5	RCC	2.3	Sunitinib	0
20 GF	F	43.5	CRC	0	Sorafenib	3
21 AL	F	54.3	RCC	0	Sunitinib	0
22 AR**	M	60.0	RCC	0	Sunitinib	0
23 NM	M	49.9	RCC	1.6	Sunitinib	0
24 GR*	M	51.7	Thyroid	15.7	Vandetanib	0
25 DC	M	49.2	CRC	0	Sorafenib	3
26 VJL*	M	56.4	Thyroid	36.9	Sorafenib	0
27 AJ*	M	32.4	STS	5.7	Sorafenib	3
28 MM	F	55.4	STS	135.1	Sorafenib	1
29 TG	M	61.1	Thyroid	0	Sorafenib	0
30 FA*	M	24.6	RCC	11.7	Sunitinib	0
31 MP*	M	75.4	RCC	21.4	Sunitinib	0
32 TA*	F	58.0	RCC	0	Sunitinib	0
33 MB*	M	49.9	RCC	0	Sunitinib	0

[1)]CRC: Colorectal Cancer
[2)]STS: Soft Tissue Sarcoma
[3)]NA: Not Available
[4)]ACC: Adrenal Cortical Carcinoma
[5)]Thyroid: Differentiated thyroid carcinoma
[6)]RCC: Renal Cell Carcinoma
[7)]HCC: Hepatocellular Carcinoma
Patients with * were treated by 2 MTKIs; Patients with ** were treated by more than 2 MTKIs.

Supplementary Table S2: Overview of 7 multi-kinase inhibitors (MTKI) and their targets

Inhibitor name	Marketed name	Reported Targets
**Sorafenib**	Nexavar	BRAF, RAF1, VEGFR3 (FLT4), VEGFR2(KDR), FLT3, PDGFRβ, KIT, RET, VEGFR1(FLT1), FGFR1
**Sunitinib**	Sutent	VEGFR1(FLT1), KIT, VEGFR2(KDR), VEGFR3(FLT4), FLT3, CSF1R, PDGFRα, PDGFRβ
**Pazopanib**	Votrient	VEGFR1(FLT1), VEGFR2(KDR), VEGFR3(FLT4), PDGFRα, PDGFRβ, KIT, FGFR3, ITK/TSK, FGF1, SH2B3
**Regorafenib**	Stivarga	RET, VEGFR1(FLT1), VEGFR2(KDR), VEGFR3(FLT4), KIT, PDGFRα, PDGFRβ, FGFR1, FGFR2, angiopoietin-1 receptor(TEK), DDR2, High affinity nerve growth factor receptor(NTRK1), EPHA2, RAF1, BRAF, MAPK11, FRK, ABL1
**Axitinib**	Inlyta	VEGFR1(FLT1), VEGFR2(KDR), VEGFR3(FLT4), KIT, PDGFRβ
**Vandetanib**	Caprelsa	VEGFR2(KDR), VEGF3(FLT4), VEGFA, EGFR, Protein-tyrosine kinase 6 (PTK6), angiopoietin-1 receptor(TEK), RET
**Cabozantinib**	Cometriq	MET, VEGFR2(KDR), RET

• CSF1R: macrophage colony-stimulating factor 1 receptor; DDR2: discoidin domain-containing receptor 2; EPHA2: Ephrin Type-A Receptor 2; FGFR: fibroblast growth factor receptor; FGF1: fibroblast growth factor 1;FRK: Tyrosine-protein kinase; ITK/TSK: IL-2 inducible T-cell kinase; MAPK11: mitogen-activated protein kinase 11; MET: Hepatocyte growth factor receptor; SH2B3: SH2B adaptor protein 3; VEGFA: vascular endothelial growth factor A;

Supplementary Table S3: List of analyzed mutation status by NGS

NGS panel

ABL1	CSF1	IGF1R	MET	PIK3CA	ROS1	SRC	VEGFR1
AKT1	CSF1R	JAK2	MPL	PIK3R1	RYK	STK11	VEGFR2
AKT2	DDB2	JAK3	MST1R	PTCH	SDHAF2	TEK	VEGFR3
ALK	DDR1	KIT	mTOR	PTEN	SDHB	TIE1	VHL
APC	DDR2	KRAS	MUSK	RB1	SDHC	TP53	
AXL	EGFR	NRAS	PDGFA	RET	SDHD	TSC1	
BRAF	ERBB2	HRAS	PDGFRA	ROR1	SMARCB1	TSC2	
CRAF	FLT3	MERTK	PDGFRB	ROR2	SMO	TYRO3	

Supplementary Table S4: Details of SCNAs on target genes and predicted sensitivity

Patients	Tumor	MTKIs	Best Response	TTC[8]	TTL[9]	SUMSCAN Group
**Discovery Cohort**						
1 BR	CRC[1]	Regorafenib	SD[7]	14	0	/
2 LJ	CRC	Regorafenib	PR[7]	6	0	/
3 CB	CRC	Regorafenib	SD	6	1	/
4 DH	CRC	Regorafenib	SD	9	2	/
5 CP	CRC	Regorafenib	SD	5	0	/
6 BZ	CRC	Regorafenib	SD	0	0	/
7 AM	CRC	Regorafenib	PD[7]	3	0	/
8 BM	CRC	Regorafenib	PD	2	4	/
9 MB	CRC	Regorafenib	PD	7	5	/
10 MC	CRC	Regorafenib	PD	2	0	/
11 JJ	CRC	Regorafenib	PD	4	1	/
12 RS	CRC	Regorafenib	PD	0	0	/
13 LH*	CRC	Regorafenib	PD	2	7	/
**Validation Cohort I**						
1 GN	STS[2]	Regorafenib	SD	14	0	Favorable
2 PA	STS	Regorafenib	SD	12	1	Favorable
3 GE	STS	Regorafenib	SD	7	2	Favorable
4 AS	STS	Regorafenib	PR	6	5	Favorable
5 PN	CRC	Regorafenib	PR	2	0	Favorable
6 CF	CRC	Regorafenib	PD	5	6	Unfavorable
7 PE	CRC	Regorafenib	PD	5	6	Unfavorable
8 PN	CRC	Regorafenib	PD	2	0	Favorable
9 BE	STS	Regorafenib	PD	2	2	Unfavorable
10 CJC	CRC	Regorafenib	PD	1	4	Unfavorable
11 RD	CRC	Regorafenib	PD	1	1	Unfavorable
12 NA	CRC	Regorafenib	PD	0	0	Unfavorable
**Validation Cohort II**						
1 VOM	ACC[3]	Sunitinib	SD	9	0	Favorable
2 NL*	STS	Sorafenib	SD	4	2	Favorable
3 DE	CRC	Sorafenib	SD	3	0	Favorable
4 DBO*	STS	Sorafenib	SD	3	1	Favorable
5 MC*	Thyroid[4]	Sorafenib	SD	3	1	Favorable
6 CC	RCC[5]	Sunitinib	PR	3	0	Favorable
7 DP*	RCC	Sunitinib	SD	3	2	Favorable
8 GAM*	RCC	Sunitinib	SD	3	0	Favorable

(*Continued*)

Patients	Tumor	MTKIs	Best Response	TTC[8)]	TTL[9)]	SUMSCAN Group
9 PJ**	Thyroid	Vandetanib	SD	3	0	Favorable
10 TM	HCC[6)]	Sorafenib	SD	2	0	Favorable
11 BR*	Thyroid	Cabozantinib	PR	2	0	Favorable
12 BJM	RCC	Axitinib	SD	2	1	Favorable
13 GS	HCC	Sorafenib	SD	2	0	Favorable
14 PF*	HCC	Sorafenib	SD	1	1	Unfavorable
15 CC	Head&neck	Pazopanib	SD	1	0	Unfavorable
16 JD*	STS	Sorafenib	SD	0	2	Unfavorable
17 DG*	Thyroid	Sorafenib	PR	0	0	Unfavorable
18 AJ	HCC	Sorafenib	PR	0	4	Unfavorable
19 RJ*	RCC	Sunitinib	CR[7)]	0	0	Unfavorable
20 GF	CRC	Sorafenib	SD	2	0	Unfavorable
21 AL	RCC	Sunitinib	PD	3	0	Unfavorable
22 AR**	RCC	Sunitinib	PD	3	0	Unfavorable
23 NM	RCC	Sunitinib	PD	3	6	Unfavorable
24 GR*	Thyroid	Vandetanib	PD	1	2	Unfavorable
25 DC	CRC	Sorafenib	PD	0	0	Unfavorable
26 VJL*	Thyroid	Sorafenib	PD	0	0	Unfavorable
27 AJ*	STS	Sorafenib	PD	0	3	Unfavorable
28 MM	STS	Sorafenib	PD	0	3	Unfavorable
29 TG	Thyroid	Sorafenib	PD	0	2	Unfavorable
30 FA*	RCC	Sunitinib	PD	0	3	Unfavorable
31 MP*	RCC	Sunitinib	PD	0	0	Unfavorable
32 TA*	RCC	Sunitinib	PD	0	1	Unfavorable
33 MB*	RCC	Sunitinib	PD	0	0	Unfavorable
**Validation cohort III**						
1 PJ	Thyroid	Sunitinib	PR	6	0	Favorable
2 PJ	Thyroid	Sorafenib	SD	6	0	Favorable
3 DBO	STS	Regorafenib	SD	5	1	Favorable
4 NL	STS	Pazopanib	SD	5	1	Favorable
5 LP	GIST	Pazopanib	SD	4	1	Favorable
6 BR	Thyroid	Vandetanib	PR	3	0	Favorable
7 LP	GIST	Regorafenib	SD	2	4	Unfavorable
8 PF	HCC	Regorafenib	SD	2	1	Favorable
9 DP	RCC	Axitinib	SD	2	1	Favorable
10 MC	Thyroid	Pazopanib	SD	2	0	Favorable
11 GAM	RCC	Axitinib	SD	2	0	Favorable
12 DG	Thyroid	Pazopanib	SD	0	0	Unfavorable

(*Continued*)

Patients	Tumor	MTKIs	Best Response	TTC[8)]	TTL[9)]	SUMSCAN Group
13 LP	GIST	Sunitinib	PD	3	2	Favorable
14 TH	CRC	Regorafenib	PD	1	0	Unfavorable
15 CJC	CRC	Sorafenib	PD	1	1	Unfavorable
16 AJ	STS	Pazopanib	PD	1	1	Unfavorable
17 LH	CRC	Sorafenib	PD	0	0	Unfavorable
18 AR	RCC	Sorafenib	PD	0	0	Unfavorable
19 JD	STS	Pazopanib	PD	0	1	Unfavorable
20 VJL	Thyroid	Pazopanib	PD	0	0	Unfavorable
21 GR	Thyroid	Sunitinib	PD	0	8	Unfavorable
22 FA	RCC	Axitinib	PD	0	2	Unfavorable
23 AR	RCC	Axitinib	PD	0	0	Unfavorable
24 TA	RCC	Axitinib	PD	0	0	Unfavorable
25 RJ	RCC	Axitinib	PD	0	0	Unfavorable
26 MB	RCC	Axitinib	PD	0	0	Unfavorable

[1)]HCC: Hepatocellular Carcinoma
[2)]STS: Soft Tissue Sarcoma
[3)]ACC: Adrenal Cortical Carcinoma
[4)]Thyroid: Differentiated thyroid carcinoma
[5)]RCC: Renal Cell Carcinoma
[6)]HCC: Hepatocellular Carcinoma
[7)]CR: Complete Response; PR: Partial Response; PD: Progressive Disease; SD: Stable Disease
[8)]TTC: Tumor Target Charge (All gain events were considered equally)
[9)]TTL: Tumor Target Loss (All lost events were considered equally)
Patients with * were treated by 2 MTKIs; Patients with ** were treated by more than 2 MTKIs.

Supplementary Table S5: Patients Characteristics and details of SCNAs and mutations on target genes

No°	MTKIs	Tumor	Best response	TTC[1)]	TTC Details	TTL[2)]	TTL Details	Mutation of target genes
**Validation Cohort I**								
**1 GN**	Regorafenib	sarcoma	SD[3)]	14	NTRK1, DDR2, RAF1, KIT, KDR, PDGFRα, PDGFRβ, FLT4, BRAF, FGFR1, TEK, ABL1, FLT1, MAPK11	0	/	None
**2 PA**	Regorafenib	sarcoma	SD	12	NTRK1, DDR2, RAF1, KIT, KDR, PDGFRα, PDGFRβ, FLT4, BRAF, FGFR1, TEK, ABL1	1	MAPK11	None
**3 GE**	Regorafenib	Sarcoma	SD	6	EPHA2, NTRK1, DDR2, FLT4, BRAF, FGFR1, ABL1	4	TEK, RET, FGFR2, MAPK11	None
**4 AS**	Regorafenib	Sarcoma	PR[3)]	6	NTRK1, DDR2, RAF1, KIT, KDR, PDGFRα	5	FGFR1, TEK, RET, FGFR2, MAPK11	None
**5 PN**	Regorafenib	CRC[4)]	PR	2	FGFR1, FLT1	0	/	None
**6 CF**	Regorafenib	CRC	PD[3)]	6	RAF1, FRK, BRAF, TEK, RET, FLT1	6	EPHA2, KIT, KDR, PDGFRα, PDGFRβ, FLT4	None
**7 PE**	Regorafenib	CRC	PD	5	PDGFRα, FGFR1, RET, FGFR2, FLT1	6	EPHA2, PDGFRβ, FLT4, BRAF, TEK, MAPK11	None
**8 PN**	Regorafenib	CRC	PD	2	BRAF, FLT1	0	/	None
**9 BE**	Regorafenib	Sarcoma	PD	2	PDGFRβ, FLT4	2	TEK, FLT1	None
**10 CJC**	Regorafenib	CRC	PD	1	BRAF	4	FGFR1, RET, FGFR2, MAPK11	None
**11 RD**	Regorafenib	CRC	PD	1	FLT1	1	MAPK11	None
**12 NA**	Regorafenib	CRC	PD	0	/	0	/	None

(*Continued*)

No°	MTKIs	Tumor	Best response	TTC[1)]	TTC Details	TTL[2)]	TTL Details	Mutation of target genes
**Validation Cohort II**								
**1 VOM**	Sunitinib	ACC[5)]	SD	9	KIT, KDR, PDGFRα, CSF1R, PDGFRβ, FLT4, RET, FLT1, FLT3	0	/	None
**2 NL***	Sorafenib	Sarcoma	SD	4	KIT, KDR, FLT4, BRAF	2	FLT1, FLT3	None
**3 DE**	Sorafenib	CRC	SD	3	BRAF, FLT1, FLT3	0	/	None
**4DBO***	Sorafenib	Sarcoma	SD	3	RAF1, RET, FGFR1	1	BRAF	None
**5 MC***	Sorafenib	Thyroid[6)]	SD	3	PDGFRβ, FLT4, BRAF	1	RAF1	None
**6 CC**	Sunitinib	RCC[7)]	PR	3	CSF1R, PDGFRβ, FLT4	0	/	None
**7 DP***	Sunitinib	RCC	SD	3	CSF1R, PDGFRβ, FLT4	2	FLT1, FLT3	None
**8 GAM***	Sunitinib	RCC	SD	3	CSF1R, PDGFRβ, FLT4	0	/	None
**9 PJ****	Vandetanib	Thyroid	SD	3	FLT4, EGFR, PTK6	0	/	None
**10 TM**	Sorafenib	HCC[8)]	SD	2	PDGFRβ, FLT4	0	/	None
**11 BR***	Cabozantinib	Thyroid	PR	2	HGF, RET	0	/	RET 918M > TM
**12 BJM**	Axitinib	RCC	SD	2	PDGFRβ, FLT4	1	FLT1	None
**13 GS**	Sorafenib	HCC	SD	2	PDGFRβ, RET	0	/	None
**14 PF***	Sorafenib	HCC	SD	1	BRAF	1	RAF1	None
**15 CC**	Pazopanib	Head&neck	SD	1	SH2B3	0	/	None
**16 JD***	Sorafenib	Sarcoma	SD	0	/	3	FGFR1, FLT1, FLT3	None
**17 DG***	Sorafenib	Thyroid	PR	0	/	0	/	DDR2 585M > MV
**18 AJ**	Sorafenib	HCC	PR	0	/	4	RAF1, BRAF, FLT1, FLT3	None
**19 RJ***	Sunitinib	RCC	CR[3)]	0	/	0	/	None
**20 GF**	Sorafenib	CRC	SD	2	FLT1, FLT3	0	/	None

(*Continued*)

No°	MTKIs	Tumor	Best response	TTC[1)]	TTC Details	TTL[2)]	TTL Details	Mutation of target genes
**21 AL**	Sunitinib	RCC	PD	3	CSF1R, PDGFRβ, FLT4	0	/	None
**22 AR****	Sunitinib	RCC	PD	3	CSF1R, PDGFRβ, FLT4	0	/	None
**23 NM**	Sunitinib	RCC	PD	3	CSF1R, PDGFRβ, FLT4	6	KIT, KDR, PDGFRα, RET, FLT1, FLT3	None
**24 GR***	Vandetanib	Thyroid	SD	1	PTK6	2	KDR, FLT4	None
**25 DC**	Sorafenib	CRC	PD	0	/	0	/	FLT4 non COSMIC
**26 VJL***	Sorafenib	Thyroid	PD	0	/	0	/	BRAF V600E
**27 AJ***	Sorafenib	Sarcoma	PD	0	/	3	RAF1, FLT1, FLT3	None
**28MM**	Sorafenib	Sarcoma	PD	0	/	3	RET, FLT1, FLT3	None
**29 TG**	Sorafenib	Thyroid	PD	0		2	FLT1, FLT3	None
**30 FA***	Sunitinib	RCC	PD	0	/	3	KIT, KDR, PDGFRα	None
**31 MP***	Sunitinib	RCC	PD	0	/	0	/	None
**32 TA***	Sunitinib	RCC	PD	0	/	1	RET	None
**33 MB***	Sunitinib	RCC	PD	0	/	0	/	None
**Validation Cohort III**								
**1 PJ**	Sunitinib	Thyroid	PR	6	CSF1R, PDGFRβ, FLT4, RET, FLT1, FLT3	0	/	FLT3 680A > AV, FLT3 330C > YC, FLT3 333S > SL
**2 PJ**	Sorafenib	Thyroid	SD	6	PDGFRβ, FLT4, BRAF, RET, FLT1, FLT3	0	/	FLT3 680A > AV, FLT3 330C > YC, FLT3 333S > SL

(Continued)

No°	MTKIs	Tumor	Best response	TTC[1)]	TTC Details	TTL[2)]	TTL Details	Mutation of target genes
**3 DBO**	Regorafenib	Sarcoma	SD	5	DDR2, RAF1, FGFR1, RET, MAPK11	1	BRAF	None
**4 NL**	Pazopanib	Sarcoma	SD	5	KIT, KDR, PDGFRα , FLT4, SH2B3	1	FLT1	None
**5 LP**	Regorafenib	Sarcoma	SD	2	PDGFRβ, FLT4	4	EPHA2, TEK, ABL1, FLT1	KIT exon11
**6 BR**	Vandetanib	Thyroid	PR	3	FLT4, EGFR, PTK6	0	/	None
**7 LP**	Pazopanib	Sarcoma	SD	4	PDGFRβ, FLT4, ITK, FGF1	1	FLT1	KIT exon 11
**8 PF**	Regorafenib	HCC	SD	2	DDR2, BRAF	1	RAF1	None
**9 DP**	Axitinib	RCC	SD	2	PDGFRβ, FLT4	1	FLT1	None
**10 MC**	Pazopanib	Thyroid	SD	2	PDGFRβ, FLT4	0	/	None
**11 GAM**	Axitinib	RCC	SD	2	PDGFRβ, FLT4	0	/	None
**12 DG**	Pazopanib	Thyroid	SD	0	/	0	/	None
**13 LP**	Sunitinib	Sarcoma	PD	3	CSF1R, PDGFRβ, FLT4	2	FLT1, FLT3	KIT exon 11
**14 TH**	Regorafenib	CRC	PD	1	FGFR1	0	/	None
**15 CJC**	Sorafenib	CRC	PD	1	BRAF	1	FGFR1	None
**16 AJ**	Pazopanib	Sarcoma	PD	1	PDGFRα	1	FLT1	None
**17 LH**	Sorafenib	CRC	PD	2	FLT1, FLT3	3	RAF1, KIT, KDR	None
**18 AR**	Sorafenib	RCC	PD	2	PDGFRβ, FLT4	1	RAF1	None
**19 JD**	Pazopanib	Sarcoma	PD	0	/	1	FLT1	None
**20 VJL**	Pazopanib	Thyroid	PD	0	/	0	/	None
**21 GR**	Sunitinib	Thyroid	PD	0	/	8	KIT, KDR, PDGFRα, CSF1R, PDGFRβ, FLT4, FLT1, FLT3	None
**22 FA**	Axitinib	RCC	PD	0	/	2	KDR, KIT	None
**23 AR**	Axitinib	RCC	PD	0	/	0	/	None

(*Continued*)

No°	MTKIs	Tumor	Best response	TTC[1)]	TTC Details	TTL[2)]	TTL Details	Mutation of target genes
**24 TA**	Axitinib	RCC	PD	0	/	0	/	None
**25 RJ**	Axitinib	RCC	PD	0	/	0	/	None
**26 MB**	Axitinib	RCC	PD	0	/	0	/	None

[1)]TTC: Tumor Target Charge
[2)]TTL: Tumor Target Loss
[3)]CR: Complete Response; PR: Partial Response; PD: Progressive Disease; SD: Stable Disease
[4)]CRC: Colorectal
[5)]ACC: Adrenal Cortical Carcinoma
[6)]Thyroid: Differentiated Thyroid Carcinoma
[7)]RCC: Renal cell carcinoma
[8)]HCC: Hepatocellular carcinoma
Patients with * were treated by 2 MTKIs; Patients with ** were treated by more than 2 MTKIs.
